# Emergency medicine resident crisis resource management ability: a simulation-based longitudinal study

**DOI:** 10.3402/meo.v19.25771

**Published:** 2014-12-09

**Authors:** Samuel Clarke, Timothy Horeczko, Matthew Carlisle, Joseph D. Barton, Vivienne Ng, Sameerah Al-Somali, Aaron E. Bair

**Affiliations:** 1 Department of Emergency Medicine, UC Davis School of Medicine, Sacramento, CA, USA; 2 Department of Emergency Medicine, Harbor-UCLA Medical Center, Los Angeles, CA, USA; 3 Office of Medical Education, LSU Health Sciences Center, New Orleans, LA, USA; 4 Department of Emergency Medicine, University of Arizona Medical Center, Tucson, AZ, USA; 5 Department of Emergency Medicine, King Abdulaziz Medical City, Riyadh, Saudi Arabia

**Keywords:** simulation, assessment, crisis resource management

## Abstract

**Background:**

Simulation has been identified as a means of assessing resident physicians’ mastery of technical skills, but there is a lack of evidence for its utility in longitudinal assessments of residents’ non-technical clinical abilities. We evaluated the growth of crisis resource management (CRM) skills in the simulation setting using a validated tool, the Ottawa Crisis Resource Management Global Rating Scale (Ottawa GRS). We hypothesized that the Ottawa GRS would reflect progressive growth of CRM ability throughout residency.

**Methods:**

Forty-five emergency medicine residents were tracked with annual simulation assessments between 2006 and 2011. We used mixed-methods repeated-measures regression analyses to evaluate elements of the Ottawa GRS by level of training to predict performance growth throughout a 3-year residency.

**Results:**

Ottawa GRS scores increased over time, and the domains of *leadership, problem solving*, and *resource utilization*, in particular, were predictive of overall performance. There was a significant gain in all Ottawa GRS components between postgraduate years 1 and 2, but no significant difference in GRS performance between years 2 and 3.

**Conclusions:**

In summary, CRM skills are progressive abilities, and simulation is a useful modality for tracking their development. Modification of this tool may be needed to assess advanced learners’ gains in performance.

The current zeitgeist in graduate medical education centers around a competency-based model that emphasizes individualized instruction and objective evidence of learning outcomes (1). In the United States, the Accreditation Council for Graduate Medical Education (ACGME) announced the current phase of the Outcomes Project, the Next Accreditation System (NAS), in February of 2012 (2, 3). The NAS directs residency programs to demonstrate trainees’ progressive mastery of knowledge, skills, and behaviors pertaining to the six core clinical competencies. In the field of emergency medicine (EM), simulation has been proposed as one of the key modalities for assessing residents’ clinical abilities within these newly framed ‘Milestones’ (4). The 2008 Society for Academic Emergency Medicine Consensus Conference on ‘The Science of Simulation in Healthcare’ held that ‘strong leadership skills are crucial to the practice of EM and thus should be a focus of team training’ (5). As the NAS is enacted, there is a growing need for evidence-based tools to evaluate trainees’ teamwork and interpersonal communication skills (6). Crisis resource management (CRM) refers to the constellation of cognitive and interpersonal communication skills that comprise effective team performance (7, 8).

Simulation has been well established as a means of assessing competency in a variety of individual clinical applications. However, limited evidence exists for the use of simulation to demonstrate growth of clinical competencies within individual learners over time. While the Ottawa GRS has been used to evaluate residents and other health professionals in a given (static) stage of their clinical development, there is no current literature reporting the use of the Ottawa GRS to evaluate learners’ longitudinal growth 9–14) . We hypothesized that EM residents’ non-technical skills develop in a positive and relatively linear fashion over the course of residency, and that the Ottawa GRS instrument would reflect this growth.

## Methods

This was an observational cohort study conducted at a single academic emergency medicine residency program based in Northern California. The Institutional Review Board at the UC Davis School of Medicine approved the study. Individual resident data were de-identified prior to analysis.

The Department of Emergency Medicine at UC Davis has held annual resident simulation assessments since 2006 to augment training and to satisfy ACGME requirements in resident evaluation. Residents are tested in a single session in which each resident performs one case. Residents are scored by multiple faculty raters using the Ottawa Crisis Resource Management Global Rating Scale (Ottawa GRS), a tool developed to evaluate team leadership and communication in the simulation environment (14). Simulated patient scenarios were developed via an iterative process by EM faculty with expertise in simulation education and scenario development. The scenarios were targeted in complexity to postgraduate year of training and were vetted by a consensus group of EM faculty with extensive simulation experience.

One hundred and three residents trained at the UC Davis EM program during the study period, 2006–2011. The EM residency at UC Davis is a 3-year, ACGME approved program established in 1989. UC Davis residents spend 70% of their clinical education at UC Davis Medical Center (UCDMC), an urban academic Level I trauma center with an annual emergency department census of 70,000 visits per year. Residents also train at a nearby private Level II trauma center with an annual Emergency Department census of 95,000 visits per year.

We included in our analysis only residents for whom we had complete longitudinal data during the study period (i.e., all 3 years of training). Fifty-eight residents were excluded from our study: 51 residents due to training cycle (i.e., entered the study as a PGY-2 or PGY-3 or completed the study as a PGY-1 or PGY-2), four residents entered the residency program as PGY-2 or PGY-3 and did not complete the PGY-1 assessment, two residents did not participate in the simulation assessment in their PGY-3 year, and one resident left the training program after the intern year. The remaining 45 residents qualified for the analysis.

### Study protocol

The PGY-1-level scenario involves a patient who presents with an ST-segment elevation myocardial infarction. The patient decompensates into ventricular fibrillation and requires advanced cardiac life support. The PGY-2-level scenario involves a patient in respiratory failure and septic shock who ultimately requires intubation, central venous access, and vasopressor support. The PGY-3-level scenario involves a patient with multi-system trauma who requires intubation, chest tube placement, blood transfusion, and negotiation with a difficult consultant (Appendices A, B, and C).

All simulations were conducted in November and December of each academic year during the study period. Residents managed their cases individually after a brief standardized introduction to the simulator and case stem. A METI high-fidelity Human Patient Simulator (CAE Healthcare, Quebec, Canada) was used for all scenarios. Medical and nursing students as well as emergency department nurses served as confederates in the scenarios. Confederates were instructed to follow the direction of the participating resident but were not permitted to offer any suggestions in management or clinical decision making.

Residents’ performance was rated using the Ottawa GRS Scale (Appendix D) (14). The Ottawa GRS consists of five CRM-related domains (*leadership*, *problem solving*, *situational awareness*, *resource utilization*, and *communication*) and an *overall* performance rating. Each domain is ranked on a 7-point Likert-style scale (seven being the highest), with descriptive anchors to aid in scoring. The Ottawa GRS has been previously validated and has shown strong interrater reliability and discriminative ability between PGY-1 and PGY-3 trainees (13). Scores were used to provide formative feedback to the resident as well as to identify residents in need of remediation. Twelve residents performed below expectations during at least one of the three assessments and were allowed a retest using an alternate scenario. Data from repeat testing was not included in our analysis.

Three to five EM faculty members were present for each session and directly observed the simulation exams. Each of the faculty raters evaluated the residents during the simulation and independently recorded their scores using the Ottawa GRS instrument. All assessments were performed in real time without reliance on video playback. While the number of raters during a given assessment varied, the group of faculty raters (eight in total) remained consistent throughout the study period. Each of the raters received training on the use of the Ottawa GRS instrument and each was asked to score resident performance based on the descriptive anchors embedded in the Ottawa GRS instrument.

### Outcome measures

The primary outcome of interest was to measure the interval change in resident performance by PGY level for the individual components of the Ottawa GRS. The secondary outcome of interest was to determine which elements of the Ottawa GRS were most predictive of overall performance.

### Data analysis

We evaluated mean GRS scores with standard deviations and interquartile ranges by individual over time and by PGY status.

We constructed two repeated measures models to evaluate: 1) the interval significance of individual performance within a PGY class and between classes within the institution and 2) the components of the Ottawa GRS that were predictive of overall performance. The first was a mixed-effects repeated measures model that included each component of the Ottawa GRS as a candidate predictor for interval significance of an individual's performance throughout residency. The second was a repeated-measures generalized linear mixed model, which included components of the Ottawa GRS (*leadership*, *problem solving*, *situational awareness*, *resource utilization*, *communication*, and *overall*) and interaction terms as candidate predictors of *overall* performance. The second model included a pooled analysis of all participants, controlling for PGY status. In each model, the clustered fixed effects were the individual (subject) effect and his or her PGY status. Specifications for the Ottawa GRS component model and the Ottawa GRS time-interval model are included in the corresponding tables. All analyses were performed using SAS software, version 9.3 (SAS Institute Inc., Cary, NC, USA; 2011).

## Results

**Fig. 1:**
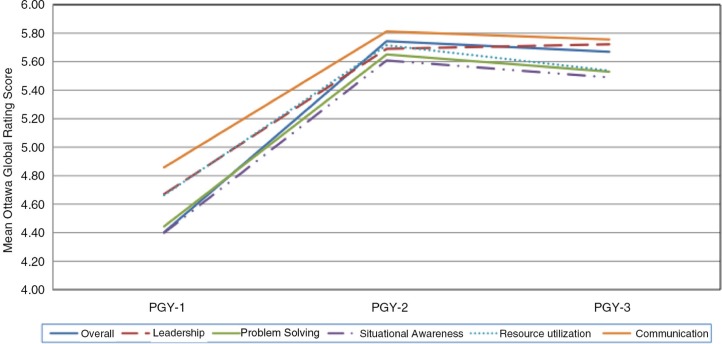
Performance by component of the Ottawa Global Rating Scale over time.

**Fig. 2:**
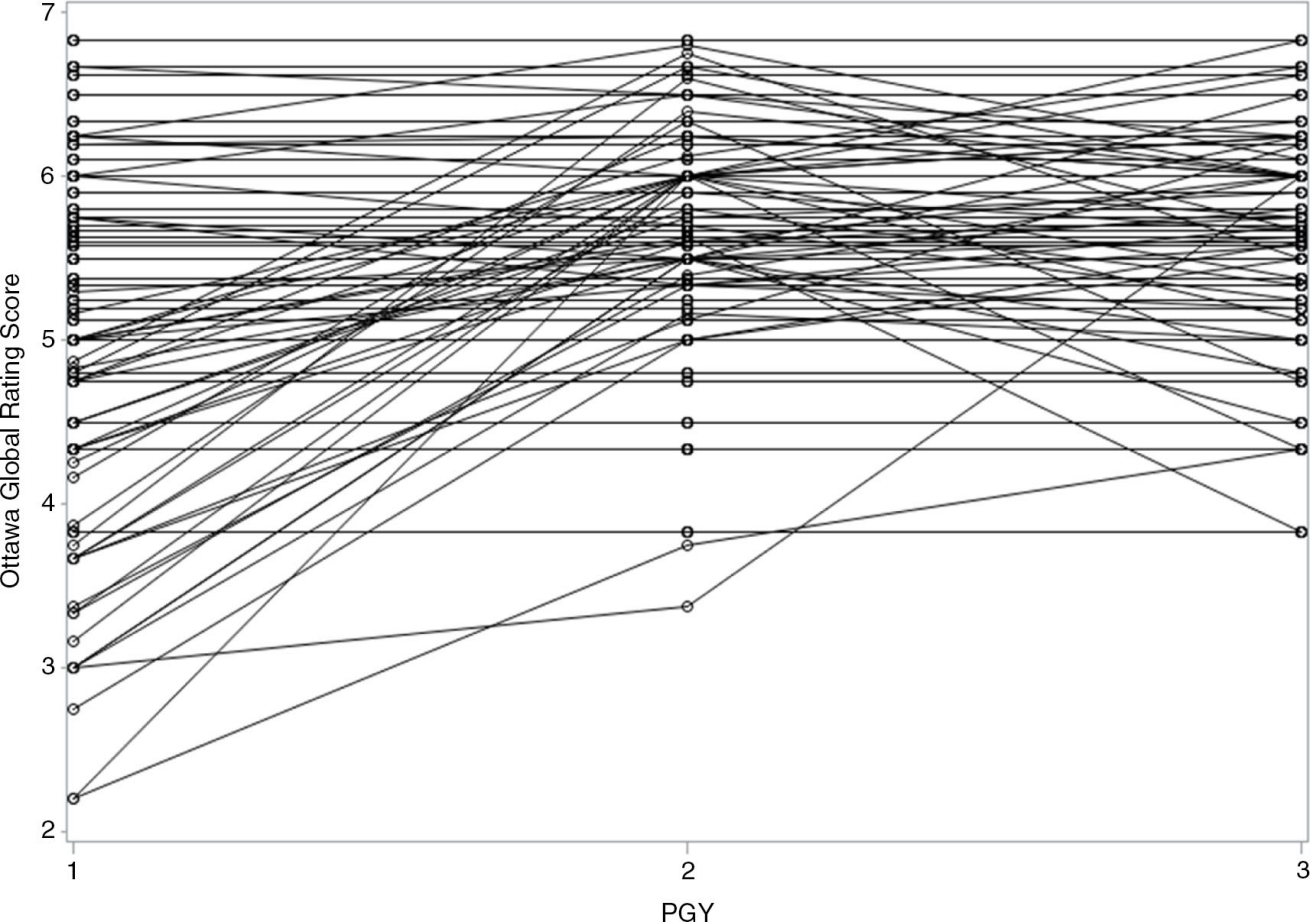
Profile plot of overall performance of each participant over time.

The demographics of the 45 residents are shown in Table 1. Mean GRS scores increased throughout each training year, with greater within-group variance for the PGY-1 level residents than in subsequent training years (Table 2). The greatest increase in mean GRS scores occurred between the PGY-1 and PGY-2 years (Fig. 1), despite individual variability in performance trajectory (Fig. 2).

**Table 1:** Resident participant characteristics, *N*=45

Class graduation year	No. of participants	Male	Female	Average age	Age range
2009	11	6	5	33	29–37
2010	12	8	4	32	29–38
2011	10	4	6	32	30–34
2012	12	9	3	32	28–41
Total	45	27	18	32	28–41

**Table 2:** Mean scores for components of the Ottawa Global Rating Scale, *N*=45

	PGY-1			PGY-2			PGY-3		
	
Component	Mean	SD	IQR	Mean	SD	IQR	Mean	SD	IQR
Overall	4.40	1.13	3.67–5.00	5.74	0.67	5.50–6.00	5.67	0.66	5.33–6.00
Leadership	4.67	0.97	4.00–5.20	5.69	0.60	5.38–6.00	5.72	0.50	5.50–6.00
Problem solving	4.44	1.15	3.67–5.17	5.65	0.66	5.38–6.00	5.53	0.64	5.25–6.00
Situational awareness	4.40	1.09	3.67–5.00	5.61	0.72	5.25–6.00	5.49	0.64	5.20–6.00
Resource utilization	4.66	0.92	3.90–5.00	5.72	0.63	5.40–6.20	5.54	0.67	5.25–6.00
Communication	4.86	0.93	4.40–5.40	5.81	0.57	5.58–6.20	5.76	0.58	5.60–6.13

The overall model demonstrated varying performance of individual components of the GRS (Table 3). *Leadership*, *problem solving*, and *resource utilization* showed statistical significance in the overall model.

**Table 3:** Model performance using components of the Ottawa Global Rating Scale

Component	*F*	*p*
Leadership	8.80	0.004
Problem solving	36.48	<0.0001
Situational awareness	0.08	0.8
Resource utilization	16.21	0.0001
Communication	0.00	1.00

Model: overall performance=β_0_+β_1_(leadership)+β_2_(problem solving)+β_3_(situational awareness)+β_4_(resource utilization)+β_5_(communication)+β_6_(PGY status)+β_7_(individual subject)+β_8–12_(interaction terms: PGY status*individual component)+Z_ij_ν_i_.

Model specifications: repeated measures generalized linear mixed models (proc glimmix); residual maximum pseudo-likelihood method; Gaussian distribution, identity link.

In the longitudinal growth model, the individual components of the Ottawa GRS – as predictors of overall performance – varied in statistical significance over time (Table 4). All components were statistically significant in the interval from PGY-1 to PGY-2. In contrast, no significant difference in performance in any GRS domain in the interval from PGY-2 to PGY-3 was detected.

**Table 4:** Interval significance of components of the Ottawa Global Rating Scale

	Interval from PGY-1 to PGY-2				Interval from PGY-2 to PGY-3			
	
Component	Estimate	SE	*t*-value	*p*	Estimate	SE	*t*-value	*p*
Overall	−0.16	0.07	−2.18	0.03	0.05	0.07	0.76	0.45
Leadership	−0.21	0.04	−5.71	<0.0001	−0.01	0.04	−0.17	0.86
Problem solving	−0.27	0.05	−5.00	<0.0001	0.03	0.05	0.56	0.58
Situational awareness	−0.36	0.07	−5.31	<0.0001	0.04	0.07	0.58	0.56
Resource utilization	−0.44	0.09	−4.86	<0.0001	0.09	0.09	0.99	0.33
Communication	−0.90	0.13	−6.78	<0.0001	0.06	0.13	0.42	0.67

Model: component=β_0_+β_1_(PGY status)+β_2_(individual subject)+β_3_(interaction term: PGY status*individual subject)+Zγ+ɛ.

Model specifications: mixed-effects repeated measures (proc mixed) by PGY status; restricted maximum likelihood method; compound symmetry covariance.

## Discussion

In this first study to evaluate critical resource management skills over time, we found that the Ottawa GRS readily identified early growth of residents’ skills. However, the Ottawa GRS was unable to discriminate scores between higher levels of training. Early learners showed considerable variance in individual scores. As training progressed, learners’ scores clustered closer together (i.e., there was a decrease in within-group variance and increase in between-group variance). The finer differences in GRS domains were not detectable in advanced learners.

Our results should be contextualized in the step-wise evolution of the current graduate medical education evaluation paradigm. In 1999, the ACGME instituted the Outcomes Project, a multi-year residency accreditation initiative based on educational outcomes and resident performance. This project was based on six identified core competencies: patient care, medical knowledge, practice-based learning and improvement, interpersonal skills and communication, professionalism, and systems-based practice (15). The ACGME convened again in 2007–2008 to identify assessment methods for graduate medical programs. The group concluded that each specialty should adopt or develop specialty-appropriate assessment tools, termed ‘Milestones’, to encourage longitudinal assessment of residents, laying the groundwork for the NAS (16).

The EM milestones were introduced in 2012 and consist of 23 areas of core knowledge (e.g., diagnosis, pharmacotherapy), skill (e.g., vascular access, airway management), and behavior (e.g., task switching, team management) common to the practice of EM (3, 17). While documenting successful completion of procedural skills can be relatively straightforward, non-technical skills are more difficult to evaluate objectively (18).

In the field of healthcare simulation, a number of different tools have been developed to assess CRM ability, a concept which originated in the aviation industry as ‘Crew Resource Management’ and which describes the non-technical skills (e.g., leadership, situational awareness, and communication) needed to successfully coordinate the behaviors of multiple individuals engaged in complex activities (19). Gaba and Deanda adopted these principles to train anesthesiologists in critical operating room scenarios (8, 20). These concepts were quickly embraced by other specialties such as EM and critical care (21). Examples of CRM assessment instruments include the ANTS scale (Anesthetists’ Non-Technical Skills), NOTECHS scale (Non-technical Skills), the OTAS (Observational Teamwork Assessment of Surgery), and the Ottawa GRS (14, 22–24) . We selected the Ottawa GRS for our study because it had been rigorously validated and is straightforward and intuitive to use.

The goal of our study was to adopt a CRM assessment tool intended for discrete simulation scenarios and to use it as a ‘growth chart’ of CRM ability for individual trainees in the real-world setting of a residency program. In this novel approach, we found a statistically significant difference in performance across all components of the Ottawa GRS between PGY-1 and PGY-2 levels. However, no significant differences were found in performance on any component of the Ottawa GRS between PGY-2 and PGY-3 levels.

Our findings may be explained through the trainee's own perspective. First-year residents come to an institution with varying backgrounds, strengths, and weaknesses. The within-group variance seen in earlier stages later diminishes, likely due to the natural course of training in the same environment. As learners ‘homogenize’ through their training, the Ottawa CRM loses discriminative ability to detect progression through domains. This is consistent with the study by Kim et al. (14) which reported that the GRS instrument had good discriminative ability when comparing PGY-1 residents to PGY-3 residents in two standardized emergency scenarios.

Our study has a number of important limitations. It was conducted at a single residency program and included a limited number of participants in a single site, affecting the generalizability of our observed results. Raters were faculty within our department and therefore not blinded to resident training level and prior clinical performance. The data were obtained over the course of multiple years by a small but variable group of faculty raters, as all raters were not present for every scenario. Individual rater data were not tracked in the database. Although interrater reliability for a particular encounter could not be evaluated, the Ottawa GRS has previously been shown to demonstrate good interrater reliability (14). While the raters did not receive extensive training in the use of the Ottawa GRS instrument, they were well versed in its rankings and appropriate use; in addition, the investigators felt the tool was sufficiently intuitive given its use of descriptive anchors to provide a reasonably reproducible score on an individual encounter.

Other institution-specific factors likely influenced our findings. The scenarios were honed over multiple years preceding the study and were targeted to each PGY level; however, they were not originally designed specifically to evoke responses for discrete GRS domains. The relatively high mean scores of residents at all levels of training suggests a possible ceiling effect that obscures true differences in ability in the advanced stages of residency (i.e., the interval between PGY-2 and PGY-3). This leniency bias may be secondary to generational differences in the student–teacher relationship, inadequate rater training, or inherent properties of global rating scales in resident assessment (25, 26) – all features with which many programs struggle.

Finally, our study design was vulnerable to a number of confounders commonly encountered in education studies. Given the frequency with which our residents are exposed to simulation, it is possible that the gains observed in residents’ CRM ability are attributable in part to increased confidence and facility with simulation itself, rather than with the material tested. Likewise, although participants were instructed not to share details of the scenarios with each other, some degree of “contamination” between groups was unavoidable.

## Conclusions

Our study supports the use of the Ottawa GRS to demonstrate progression of CRM ability in individual learners over the course of residency training in EM. Specifically, the Ottawa GRS instrument shows promise as a tool for charting growth early in training but may need modification for use in advanced learners. These findings may benefit the design of a future prospective, multicenter, multi-domain observational study of residents’ attainment of developmental milestones in EM.

## Figures and Tables

**Figure:**
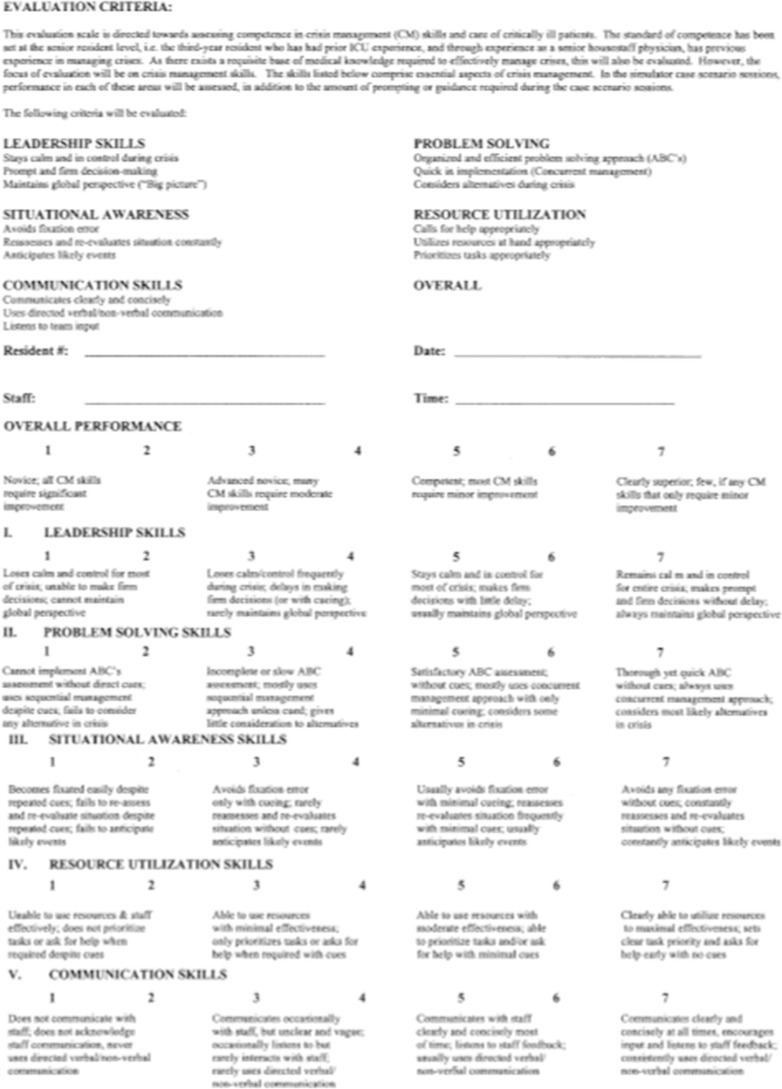
Ottawa Crisis Resource Management Global Rating Scale (Ottawa GRS)

## Appendix A

**Table:** PGY-1 Simulation Case

Case details VF (R1)	Goals	Critical actions
65-year-old male BIBA c/o CP for 2 h Intermittent CP for 3 weeks ‘Feels like my first MI’ Onset upon waking, 10/10 Substernal, left arm & jaw, nausea. Woke up with this pain, now 5/10 PMH: MI, DM, HTN, Chol Meds: Metformin, Lipitor, Maxzide PSH: Left total knee (years ago) FSH: CAD; 40 pk yrs, married, retired NKDA	Identify that the CP patient is a priority patient Rapidly assess the potentially critical patient (Medical Red) Recognize ‘typical’ cardiac ischemia symptoms Obtain history Elicit drug allergies Get EMS report Identify as priority patient (get nursing and tech support)	Perform focused physical exam Obtain 12-lead ECG Place on oxygen Start IVs Place on monitor (including SpO2)
PE: 160/90 110 20 100% on 4L NC Awake/alert, anxious, diaphoretic Lungs clear Heart tachy, regular, no murmur Perfusion good Abdomen soft EMS: CP protocol Given NTG 0.4 mg SL×3 CP 10/10→6/10 BP 190/110→150/90 Patient refused ASA (GI upset) 1st 12-lead: Anterior STEMI	Identify cardiac ischemia/infarction Differentiate medication intolerance from true allergy Recognize the need for rapid intervention in ACS/STEMI	Portable CXR Administer appropriate meds: ASA, NTG, B-blocker, morphine, heparin Arrange Cath or thrombolytics Reassess after interventions (pain, BP, heart rate)
Patient becomes unresponsive Eyes roll back No movement (V. Fib on monitor)	Identify pulseless arrest Differentiate VF from stable rhythms Assume leadership role directing ‘code’ Recognize VF requires rapid intervention (defibrillation) Use the correct ACLS algorithm for pulseless rhythms Correctly administer CPR	Start CPR immediately Appropriate defibrillation Provide a BLS airway Resume CPR immediately after shock (for 2 min. or 5 cycles) Appropriate medications: EPI or VP during compressions
CPR stops and patient remains in VF No pulse Patient is ashen and mottled Pulse oximeter flat Vomitus in the airway	Recognize pulseless rhythm Recognize shockable rhythm Recognize the need for advanced airwa	Intubate & confirm tube placement Appropriate ongoing CPR and shocks Appropriate antiarrhythmic (i.e., lidocaine or amiodarone)
CPR stops and SR ensues 100/50 120 94% with bag Patient agitated	Recognize ROSC Recognize hypoxemia as dangerous in coronary ischemia	Reassess condition Post intubation management Portable CXR End scenario

## Appendix B

**Table:** PGY-2 Simulation Case

Case details Sepsis (R2)	Goals	Critical actions
48-year-old male presents with 3 days of cough, fever, green sputum, and 1-day dyspnea. CP is pleuritic. He has mild orthostatic symptoms. ROS negative except as above PMH: Previously healthy, no surgeries. No Meds F/SH: Tobacco 30 pack years, Etoh – ‘a fair amount’, no illicit drug use. Married, monogamous. Travels extensively in US for work (sales). 3 school aged children – all have URIs. NKDA	Meets SIRS criteria Obtain history Treat patient as priority	Pulse oximetry Order CXR
PE: HR 100 BP 110/70 RR28 T 38.2 92% on RA, 100% on NRB Awake & alert Has accessory muscle use Moist cough Crackles at R base *CXR shows Right Lower Lobe Pneumonia*	Sepsis=SIRS plus documented infection Identify PNA Order Antibiotics Oxygen Labs – CBC/Chem7/lactate/ABG Fluid bolus	Identify PNA Order Appropriate antibiotics Oxygen
20 min later: Patient confused VS: HR 120 BP 90/38 RR 30 SaO2 89% on RA, 97% on NRB Labs: Na 140 K 4.5 Cl 103 HCO3-17 BUN-19 creat-1.7 glu-121, Lactate 3 WBC 20 Hct 37	SEVERE sepsis=sepsis plus at least one sign of organ hypoperfusion/dysfunction – include AMS and ↑lactate Track urine output Frequent VS check	Identify metabolic acidosis Respiratory support intubation OK, BiPAP still OK Initiate resuscitation with crystalloid Notify MICU
40 min later: Patient very confused, not compliant After 2 L bolus: HR 126 BP 90/32 RR 40 Sats 92% on NRB, 81% on RA Further 2 (4 L total) HR 130 BP 85/25	Need to recognize indication for intubation Crystalloid fluid bolus 40–60 mL/kg (i.e., 3–5 L) Foley Ventilate at 6–7 mL/kg ~ 400 mL tidal volume, PEEP 5 Consider inotropic support - Dopamine 5 mg/kg/min - Epinephrine/Norepinephrine 0.25 mg/kg/min Transfusion to keep Hct>30% Failure to respond to fluid bolus=septic shock	Intubate using appropriate technique More fluid to reach 50 mL/kg bolus Place central venous cath for vasopressors and Scv0_2_ Inotropic support
Inotropic support started Lactate 6.7 MICU busy ‘will be down soon’ Hemodynamic monitoring	Aim CVP 8–12 mm Hg Aim MAP >65, ≤90 Aim ScvO2 >70%	Post intubation management End scenario

## Appendix C

**Table:** PGY-3 Simulation Case

Case details Poly trauma (R3)	Goals	Critical actions
40-year-old male BIBA sp MVC hypotensive w/AMS Had c/o of head, chest and abdominal pain. Unrestrained driver in high speed MVC. +LOC. SBP 80 at scene. PMH/PSH: unable to provide	Rapidly assess and address multiple potential causes of hypotension in polytrauma patient	Obtain history Perform focused physical exam ABCDE and address issues Start IVs and NS bolus Place on monitor (including SpO2)
PE: 110/70 110 30 100% on 4 L NC Somnolent, intermittently, follows commands Lungs clear, abrasion across left chest Heart-tachycardia, regular, no murmur Strong pulses Abdomen soft with mild diffuse TTP and mid abrasion	Recognize need for rapid intervention Identify: Closed head injury (with dec LOC) Small, left pneumothorax Hemoperitoneum with splenic rupture (per CT)	Reassess after initial fluid bolus Appropriate prelim imaging: CXR, pelvis, FAST (+ free fluid)
Patient becomes poorly responsive and hypotensive. 90/45	Identify change	Check responsiveness Intubate to protect airway in anticipation of transport (CT/OR) Appropriate use of RSI (and dosing of agents)
No palpable blood pressure after intubation	Recognize potential causes of post intubation hypotension Exclude esophageal intubation Tension ptx Med effect Ongoing hemorrhage	Appropriate management of post intubation hypotension Appropriate management of pneumothorax in concert with intubation Appropriate use of blood products
VS SBP 90/p 110 Pt will require transfer to trauma center after initial stabilization of intraabdominal injuries	Prioritize disposition	Contact surgeon at receiving center Contact local surgeon for laparotomy to stabilize for transport End scenario

## Appendix D

Reproduced with permission from Lippincott Williams & Wilkins.
